# Neuroinflammation in Epilepsy: Biochemical and Molecular Mechanisms and Implications for Natural Product-Driven Drug Discovery

**DOI:** 10.3390/ijms27135857

**Published:** 2026-06-29

**Authors:** Arthur Lins Dias, Pablo R. da Silva, Livia R. P. Souza, Hugo F. O. Pires, Maria C. F. Gonçalves, Luiza C. D. Neri, Nayana M. M. V. Barbosa, André Luiz Leocádio de Souza Matos, Anuraj Nayarisseri, Marcus T. Scotti, Adriana M. F. de Oliveira-Golzio, Cícero F. B. Felipe, Mirian Graciela da Silva Stiebbe Salvadori, Luciana Scotti

**Affiliations:** 1 Institute of Drugs and Medicines Research, Federal University of Paraíba, João Pessoa 58051-900, Brazil; arthurlinsd@gmail.com (A.L.D.); pablorayff@ltf.ufpb.br (P.R.d.S.); lrps@academico.ufpb.br (L.R.P.S.); hugofernandes@ltf.ufpb.br (H.F.O.P.); mcfg@ltf.ufpb.br (M.C.F.G.); luiza.neri@academico.ufpb.br (L.C.D.N.); nayanamedeiros@ltf.ufpb.br (N.M.M.V.B.); andre.leocadio@academico.ufpb.br (A.L.L.d.S.M.); adrianamfoliveira@gmail.com (A.M.F.d.O.-G.); cicero@dbm.ufpb.br (C.F.B.F.); mirian.salvadori@gmail.com (M.G.d.S.S.S.); 2 Postgraduate Program in Cognitive and Behavior Neuroscience, Federal University of Paraíba, João Pessoa 58051-900, Brazil; 3 Postgraduate Program in Natural and Synthetic Bioactive Compounds, Federal University of Paraíba, João Pessoa 58051-900, Brazil; mtscotti@gmail.com; 4 In Silico Research Laboratory, Eminent Biosciences, Indore 452010, India; anuraj@eminentbio.com

**Keywords:** epilepsy, neuroinflammation, natural products, drug discovery

## Abstract

Epilepsy is a chronic neurological disorder prevalent worldwide, characterized by recurrent episodes of epileptic seizures. The primary current treatment approach is pharmacological, aimed at reducing the intensity and frequency of seizures, though it does not provide a cure. Neuroinflammation plays a central role in epilepsy by activating glial cells and stimulating the release of inflammatory mediators, further disrupting the balance between excitation and inhibition, thereby promoting the onset and recurrence of seizures. Furthermore, persistent inflammatory processes induce synaptic remodeling and the formation of dysfunctional neural circuits, establishing a pathological cycle in which inflammation and epileptic activity feed into each other. In this regard, natural products represent an important avenue for the discovery of new treatments. Thus, this review aimed to relate the role of the main inflammatory targets (Inflammasome/NLRP3, NF-κB, MAPK, mTOR, COX-2/PGE2, and TLR4/HMGB1) to epilepsy and to investigate in the literature natural products acting through these pathways in the treatment of epileptic seizures. Consequently, inflammatory pathways have emerged as critical targets in epilepsy, highlighting the importance of strategies capable of modulating neuroinflammatory processes. In this context, natural products stand out as promising therapeutic alternatives, given their multitarget mechanisms of action, potential to attenuate neuroinflammation and neuronal hyperexcitability.

## 1. Introduction: Epilepsy

Epilepsy is one of the most common chronic neurological disorders in the world, affecting more than 50 million people and causing social, cognitive, and economic impacts [1]. Patients with epilepsy experience recurrent seizures, characterized by signs of excessive and disordered neuronal activity, with focal, generalized, or undetermined origins, leading to a reduced quality of life and potentially being fatal [2].

Based on their origin in the brain, epileptic seizures can be classified as focal, generalized, or of unknown onset. These events can result from multiple etiological factors, including central nervous system infections, severe febrile states, genetic predisposition, and idiopathic causes, and vary in severity and in their potential to exacerbate preexisting medical conditions [3].

The pathophysiology of epilepsy involves a complex combination of anatomical, cellular, and molecular changes that contribute to the generation and propagation of epileptic seizures [4]. The most significant morphological changes associated with epilepsy are located in the hippocampus, thalamus, basal ganglia, and cerebral cortex, with mesial temporal sclerosis being one of the most prominent, particularly in temporal lobe epilepsy. This condition involves selective loss of pyramidal neurons in the CA1 and CA3 subfields, associated with reactive gliosis, leading to an imbalance between the excitatory and inhibitory systems, synaptic reorganization, and hyperexcitability [5,6].

Epilepsy is supported at the molecular level by an interconnected triad consisting of glutamatergic excitotoxicity, oxidative stress, and neuroinflammation. Glutamate dysregulation leads to the hyperactivation of receptors, particularly NMDA receptors, promoting excessive calcium influx, the activation of neurotoxic pathways, and neuronal death. Concurrently, increased production of reactive oxygen species intensifies oxidative stress, causing damage to lipids, proteins, and DNA, as well as potentiating excitotoxicity [7,8].

In recent decades, new studies have indicated that epileptic seizures are able to activate glial cells and induce the release of inflammatory mediators, contributing to structural and functional changes in nervous tissue and to the maintenance of neuronal hyperexcitability [9]. Furthermore, neuroinflammation plays a central role by inducing changes in the expression of ion channels and receptors on neuronal membranes, disrupting the balance between excitatory and inhibitory mechanisms and creating conditions conducive to the onset of epileptic seizures.

In addition, the persistence of the inflammatory process can induce structural changes in synaptic connections, contributing to the formation of dysfunctional neural circuits. This remodeling, combined with hyperexcitability, establishes a self-perpetuating pathological cycle in which inflammation and epileptic activity feed into one another, promoting the maintenance and progression of the disease [9,10,11].

Antiepileptic drugs (AEDs) form the cornerstone of epilepsy treatment, aiming to prevent seizures and reduce their severity by modulating excessive neuronal activity. Pharmacotherapy encompasses different generations of AEDs with distinct mechanisms of action; however, only a subset of these medications is considered first-line therapy due to their favorable safety and tolerability profiles [12]. Despite their clinical efficacy, current pharmacological treatments are unable to cure epilepsy and are primarily intended to reduce the frequency and intensity of seizures. Moreover, AEDs are often associated with adverse effects, including sedation, fatigue, nausea, and vomiting, which may negatively impact patients’ quality of life. In addition, approximately 30% of patients remain refractory to treatment, frequently requiring combination therapy and consequently facing an increased risk of drug-related side effects [13,14].

Among the main groups, benzodiazepines and barbiturates stand out, acting predominantly on the GABAergic system, promoting increased neuronal inhibition and reduced excitability. While benzodiazepines allosterically modulate GABA_A_ receptors, barbiturates prolong the opening of these channels, although they carry a higher risk of adverse effects. On the other hand, newer anticonvulsants, such as lamotrigine, levetiracetam, and topiramate, act through various mechanisms, including sodium channel blockade and modulation of the release of excitatory neurotransmitters. Despite therapeutic advances, current treatment is predominantly symptomatic, focused on reducing neuronal hyperexcitability, without promoting a cure for epilepsy [15,16,17].

In this context, the search for new antiepileptic drugs is a global effort aimed at addressing the issue of treatment-related risks, as well as the problem of drug resistance, which affects a large proportion of individuals with epilepsy. Thus, natural products represent an important source of bioactive compounds of interest for the study of antiepileptic potential [18,19]. Therefore, this study aims to review the main targets related to neuroinflammation and epilepsy and to investigate natural products acting through these pathways.

## 2. Molecular Mechanisms of Neuroinflammatory Signaling Pathways and Their Modulation by Natural Products in Epilepsy

### 2.1. Inflammasome/NLRP3

Pertaining to the class of pattern recognition receptors (PRRs), the Nod-Like Receptors (NLR) family constitutes one of the most important groups of PRRs in the inflammatory response and, for this reason, has been the focus of numerous studies. The subfamilies are classified according to the N-terminal domain and comprise four subclasses: NLRA, NLRB, NLRC, and NLRP, which, once activated, promote downstream signaling and inflammasome formation, as well as the inflammatory response [20,21].

The inflammasome consists of an arrangement of multiprotein and cytoplasmic complexes composed of a PRR, the ASC adaptor protein (apoptosis-associated granule-like protein containing a CARD), and the cysteine protease caspase-1, which are activated in response to innate immunity in the presence of pathogens. Each inflammasome is stimulated in a different way, but they share common responses, such as the autoproteolytic activation of caspase-1, which produces pro-interleukin-1β (IL-1β), pro-IL-18, and gasdermin D (GSDMD) [22].

The formation of the Nod-Like Receptor Family Pyrin Domain Containing 3 (NLRP3) inflammasome is initiated upon recognition of pathogen- and damage-associated molecular patterns (PAMPs and DAMPs), which include events such as the efflux of K^+^ and Cl^−^, mitochondrial dysfunction with the release of reactive oxygen species (ROS) and mtDNA, as well as the release of cathepsin B. In this process, NLRP3 specifically interacts with the NEK7 protein, and as activation progresses, this interaction intensifies, leading to the oligomerization of NEK7 with NLRP3 into a functional complex. Once oligomerized, the receptor recruits the ASC (apoptosis-associated granule-like protein) adaptor protein through interactions between its PYD-PYD domains. In the final assembly step, the caspase recruitment domain (CARD) of ASC binds to the CARD of pro-caspase-1, consolidating the formation of the multiprotein complex known as the NLRP3 inflammasome, which is essential for the processing of inflammatory responses [22,23].

**Figure 1:**
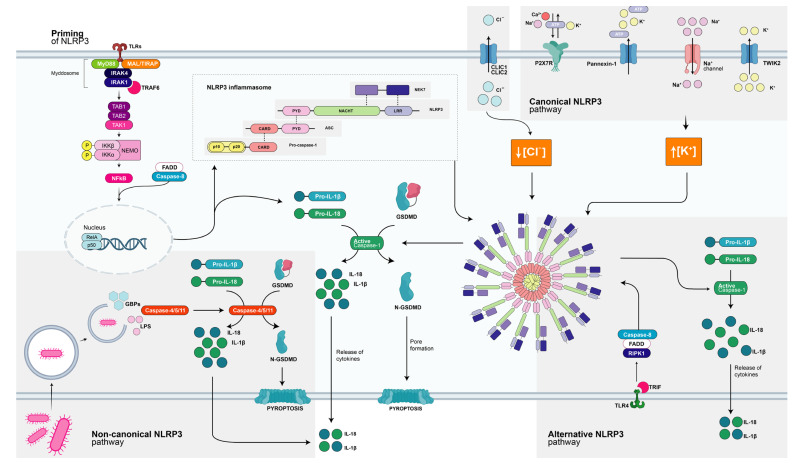
Molecular mechanisms of signaling and activation of the NLRP3 inflammasome. The process begins with the priming stage, in which PAMPs or DAMPs are recognized by TLRs, activating the MyD88 complex (composed of MyD88, IRAKs, and TRAF6), which promotes downstream signaling via NF-κB to the nucleus, resulting in the upregulation of NLRP3 expression and the transcription of pro-inflammatory cytokines from pro-IL-1β and pro-IL-18, which, via caspase-1, produce IL-1β and IL-18. Subsequent activation of NLRP3 can occur via three distinct pathways: the canonical pathway, characterized by the efflux of K^+^ and Cl^−^ ions through ion channels and receptors such as P2X7R, which promotes the oligomerization of NLRP3 with the adaptor protein ASC and pro-caspase-1; the non-canonical pathway, mediated by the recognition of cytoplasmic LPS by caspases-4 and 5 (in humans) or caspase-11 (in mice), inducing the cleavage of gasdermin D (GSDMD) and consequently leading to the process of pyroptosis. Finally, the alternative pathway, which operates via TLR4-TRIF-RIPK1 independently of ion efflux. In all scenarios, caspase-1 activation is the critical event for the formation and secretion of the pro-inflammatory cytokines IL-1β and IL-18. Note: Solid black arrows indicate activation or signaling pathways.

NLRP3 inflammasome activation can occur by three pathways: the canonical, non-canonical, and alternative pathways. The canonical pathway consists of a two-step process: priming and activation. Sensitization occurs when cytokine receptors or toll-like receptors (TLRs) recognize PAMPs or DAMPs, activating nuclear factor-kappa B (NF-κB) and its downstream signaling, thereby increasing the expression of proteins such as IL-1β, pro-caspase-1, and NLRP3. NF-κB activation occurs via the myddosome complex, a protein cluster essential for the activation and translocation of NF-κB to the nucleus and subsequent upregulation of NLRP3 (Figure 1) [24,25].

The second stage of the canonical pathway is triggered by stimuli associated with PAMPs and DAMPs. Among these stimuli, lipopolysaccharide (LPS), extracellular ATP, silica, aluminum, changes in ionic flux—such as K^+^ efflux and Ca^2+^ influx—monosodium urate (MSU) crystals, nigericin, and ROS stand out, although the mechanisms involved have not yet been fully elucidated. Recent evidence, however, indicates that human monocytes can activate the inflammasome independently of a prior sensitization step [26].

Non-canonical activation is primarily mediated by LPS, is TLR-independent, and depends on caspases-4 and 5 in humans and caspase-11 in mice. LPS reaches the cytoplasm via endocytosis or transfection and activates caspase-11, which in turn opens the pannexin 1 channel, allowing the influx of ATP and efflux of K^+^. The efflux of K^+^, as described previously, plays a key role in the activation of the NLRP3 inflammasome, as well as in the secretion of IL-1β and IL-18. Furthermore, the activation of caspase-11/4/5 promotes the cleavage of GSDMD, activating the inflammasome and inducing pyroptosis—an inflammatory form of programmed cell death involving pore formation in the membrane—via the N-terminal of GSDMD [26,27].

The alternative pathway is activated by LPS and depends on TLR4, caspase-8, FADD, and the receptor-interacting serine/threonine protein kinase 1 (RIPK1). However, it is independent of K^+^ influx, generating an inflammasome distinct from the classical NLRP3 inflammasomes arising from the canonical and non-canonical pathways, since it does not induce pyroptosis [26].

The activation of the NLRP3 inflammasome in the CNS plays a crucial role, contributing to the elimination of pathogens and promoting tissue repair processes. However, when this activation occurs over sustained periods or independently of pathogens, the accumulation of pro-inflammatory markers promotes neuroinflammation and, consequently, cell death and microglial activation to the M1 inflammatory stage, which perpetuates neuroinflammation in a cyclical manner and promotes neurodegeneration [28].

The NLRP3 inflammasome pathway is implicated in the development of epilepsy, acting as a central axis that integrates neuroinflammation, neuronal excitability, and cell death, particularly in temporal lobe epilepsy (TLE) [21,29]. Activation of this complex leads to caspase-1 cleavage and the maturation of pro-inflammatory cytokines, such as IL-1β and IL-18, whose effects are critical in modulating neuronal excitability. IL-1β, in particular, potentiates excitatory neurotransmission through positive modulation of NMDA receptors, reduces inhibitory GABAergic transmission, and increases blood–brain barrier permeability, favoring the infiltration of peripheral cells and the amplification of the inflammatory response. Complementarily, IL-18 contributes to cell recruitment and the perpetuation of inflammation, exacerbating tissue damage [30].

Seizure stimulation induces the expression of NLRP3 in both glial cells and neurons, triggering an exacerbated neuroinflammatory response that sustains epileptogenesis. In this situation, mitochondrial dysfunction and oxidative stress play a decisive role, since the accumulation of ROS, the release of mitochondrial DNA, and interaction with proteins such as TXNIP act as key signals for inflammasome activation. Experimental evidence demonstrates that pharmacological or genetic inhibition of NLRP3 reduces caspase-1 activation, the release of IL-1β/IL-18, and the occurrence of gasdermin D-mediated pyroptosis, resulting in attenuation of neuroinflammation, reduced neuronal damage, and decreased hyperexcitability. Furthermore, the improvement in mitochondrial function observed with NLRP3 inhibition reinforces the bidirectional relationship between inflammation and energy metabolism, with mitochondrial dysfunction being a factor associated with treatment resistance in epilepsy [31].

A molecular analysis of this pathway reveals that the persistence of NLRP3 inflammasome signaling extends beyond acute tissue damage, acting as a key factor in maladaptive synaptic plasticity. This process results from the continuous secretion of IL-1β mediated by active Caspase-1, which in turn promotes deleterious allosteric modulation of postsynaptic receptors through the activation of the Src tyrosine kinase. This event induces phosphorylation of the GluN2B subunit of NMDA receptors, exacerbating Ca^2+^ influx and perpetuating glutamatergic excitotoxicity, while simultaneously stimulating the internalization and reduced conductance of GABA_A_-type inhibitory receptors [30].

At the same time, oxidative stress and sustained mitochondrial dysfunction lead to chronic activation of GSDMD. The subsequent formation of pores in the cell membrane triggers selective pyroptosis of GABAergic inhibitory interneurons in hippocampal subregions, exacerbating the reduction in physiological inhibitory tone [31]. This profound structural remodeling, coupled with the degradation of the extracellular matrix via NF-κB-downstream metalloproteinase activation, establishes an autonomous hyperexcitable neuronal network. As the electrophysiological properties and basal molecular targets of the parenchyma are permanently altered by this inflammatory microenvironment, the mechanisms of action of conventional anticonvulsant drugs are impaired, as they fail to demonstrate satisfactory pharmacodynamics and therapeutic efficacy, thereby consolidating the phenotype of drug-resistant epilepsy [30,31].

In addition, NLRP3 integrates with important intracellular signaling pathways, such as NF-κB, mTOR, and MAPKs, which are involved in both the priming process and the amplification of the inflammatory response. This interaction sustains a positive feedback loop between inflammation and neuronal excitability, promoting changes in synaptic plasticity, circuit reorganization, and maintenance of the epileptic state, thereby contributing to the chronicity of the disease [23,30].

In the search for natural products for new epilepsy treatments, it has been identified that flavonoids are widely investigated in medicinal chemistry due to their potential to inhibit the propagation of seizures, especially those structurally similar to benzodiazepines, which act by modulating the GABA_A_ receptor. In this context, fisetin, a natural senolytic flavonoid, has emerged as a promising candidate for an antiepileptic drug due to its antioxidant, anti-inflammatory, and neuroprotective properties [32]. Its neuroprotective effects have already been demonstrated in several models, including arsenic- and fluoride-induced neurotoxicity [33], Alzheimer’s disease [34], and oxidative stress resulting from traumatic brain injury, involving the activation of the Nrf2/ARE pathways [35].

However, pharmacokinetic limitations, such as low water solubility, rapid metabolism, and high clearance, have driven the development of nanoformulations with the aim of optimizing their bioavailability. In this regard, a chitosan nanoparticle loaded with fisetin demonstrated, in a pilocarpine-induced temporal lobe epilepsy model (Table 1), a neuroprotective effect superior to that of the free form, evidenced by a reduction in oxidative stress, pro-inflammatory cytokines, and acetylcholinesterase activity, associated with inhibition of the ROS/TNF–NLRP3 pathway, with a consequent decrease in IL-1β and IL-18 and preservation of hippocampal neuronal integrity, crucial for controlling the feedback loop that fuels inflammatory resistance [36].

Likewise, *Curcuma longa* L., a member of the Zingiberaceae family, is an important source of bioactive compounds, especially curcumin. This curcuminoid has been extensively studied for its antioxidant, anti-inflammatory, and neuroprotective properties and is considered promising in the context of epilepsy [41]. Experimental evidence indicates that curcumin increases the activity of antioxidant enzymes, such as superoxide dismutase (SOD), catalase (CAT), and glutathione peroxidase (GPx), elevates reduced glutathione (GSH), and reduces markers of oxidative stress, such as malondialdehyde (MDA) and lipid peroxidation (Table 1) [37].

Concurrently, it modulates the inflammatory response by reducing pro-inflammatory cytokines such as tumor necrosis factor alpha (TNF) and IL-1β, as well as interfering with apoptosis-related pathways, such as the reduction of caspase-3, contributing to the preservation of hippocampal structure, especially in the CA3 region [37]. These effects are functionally reflected in a reduction in the frequency, duration, and severity of seizures, as well as an increase in seizure latency. Also noteworthy is its ability to modulate neuroinflammation by inhibiting the NLRP3 inflammasome, suggesting a relevant role in attenuating inflammation-associated neuronal excitability. Furthermore, recent evidence suggests that curcumin may potentiate therapeutic effects when used in combination with other drugs, reinforcing its multimodal profile in the management of epilepsy [42].

Resveratrol is another widely studied compound. It is a natural polyphenol with recognized antioxidant, anti-inflammatory, and neuroprotective properties (Table 1). Like other phenolic compounds, it has pharmacokinetic limitations, such as low solubility and rapid metabolism, which have led to the development of nanotechnological strategies to optimize its bioavailability in the central nervous system. From a mechanistic perspective, resveratrol acts to modulate neuroinflammation and oxidative stress by activating pathways, such as SIRT1/SIRT3. This activation results in the downregulation of NF-κB, reduced ROS production, and inhibition of NLRP3 inflammasome activation, ultimately leading to decreased release of pro-inflammatory cytokines such as IL-1β and IL-18. Furthermore, this compound promotes the modulation of microglial activation, facilitating the transition from the pro-inflammatory M1 phenotype to the anti-inflammatory M2 profile, thereby contributing to the attenuation of neuroinflammation and neuronal excitability [43].

Corroborating these findings, recent studies have developed a nucleic acid-based nanosystem with a tetrahedral structure (tFNAs), combined with a core of Prussian blue (PB) and resveratrol, capable of synergistically scavenging ROS and activating the SIRT3/SOD2 pathway, thereby increasing mitochondrial antioxidant capacity. This modulation resulted in the effective inhibition of the ROS/NLRP3 pathway and the induction of microglial polarization toward the M2 phenotype. In a kainic acid-induced epilepsy model, the system significantly reduced neuronal damage, seizure frequency, and severity, in addition to improving cognitive deficits [44].

In addition to these compounds, other natural products have also shown potential in modulating the NLRP3 inflammasome in epilepsy. Neferine, an alkaloid extracted from lotus seed embryos, exhibits anticonvulsant properties in kainic acid-induced epilepsy models. In this context, it was observed that neferine attenuates seizure activity by downregulating glutamatergic hyperactivity and the NLRP3-mediated inflammatory pathway (Table 1). The results showed reduced neuronal loss in hippocampal regions, as well as modulation of synaptic proteins such as synaptophysin and PSD95. Additionally, neuroinflammation was suppressed, with reduced gliosis, decreased production of pro-inflammatory cytokines, and reduced expression of NLRP3, caspase-1, IL-1β, and IL-18 in the hippocampus of treated animals [40]. These findings reinforce the role of natural products as modulators of the NLRP3 inflammasome pathway, highlighting their therapeutic potential in epilepsy.

### 2.2. NF-κB

NF-κB is a family of dimeric transcription factors responsible for activating approximately 500 genes associated with inflammation, B-cell maturation, and the regulation of essential cellular functions such as proliferation, growth, survival, and cell death. The common structural element among NF-κB members is the Rel homology domain (RHD), located in the N-terminal region, which is responsible for recognizing specific DNA sequences, dimerization, and interaction with inhibitory proteins of the IκB family in its inactive state [45].

Several subunits, such as RelA (p65), RelB, and c-Rel, also possess a transactivation domain (TAD) in their C-terminal region, which is essential for the activation of gene transcription. The p50 and p52 subunits lack this domain and depend on association with other proteins to exert transcriptional activity. The p105 and p100 precursors contain ankyrin-like repeats in their C-terminal region, which exert an inhibitory function by keeping NF-κB complexes in the cytoplasm until their proteolytic processing [46].

**Figure 2:**
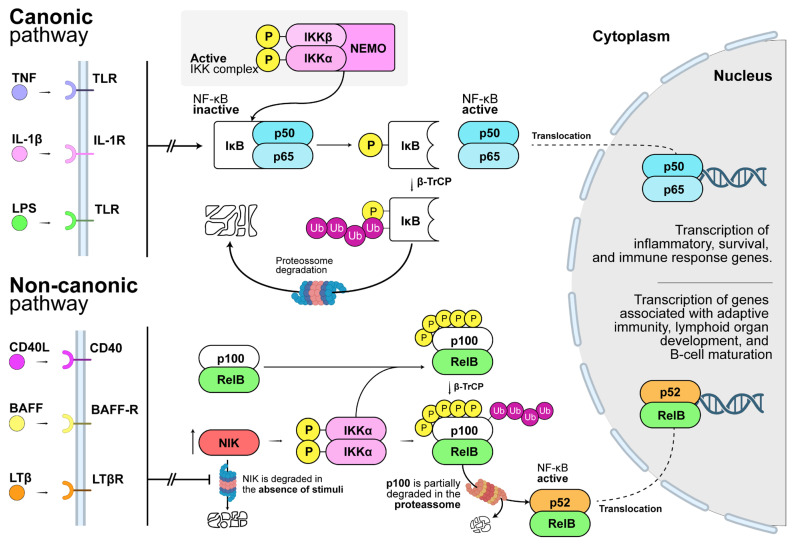
Canonical and non-canonical NF-κB activation pathways. The canonical pathway is activated by TNF, IL-1β, LPS, and ROS via the TNFR, IL-1R, and TLRs, leading to the activation of the IKK complex (IKKα, IKKβ, and NEMO) by adaptor proteins. Under basal conditions, NF-κB remains in the cytoplasm bound to IκB. The IKKβ subunit phosphorylates IκB, inducing ubiquitination by β-TrCP and subsequent proteasomal degradation. The activated p65/p50 dimers are translocated to the nucleus, where they induce the transcription of inflammatory, survival, and immune response genes. The non-canonical pathway is activated by CD40L, BAFF, and LTβ via CD40, BAFF-R, and LTβR, promoting increased levels of IKK in the cytoplasm. This increase promotes IKKα phosphorylation, which phosphorylates p100, inducing ubiquitination by β-TrCP and subsequent partial proteasomal degradation, generating the p52 subunit. p52/RelB are translocated to the nucleus, where they are responsible for the transcription of genes associated with adaptive immunity, the development of lymphoid organs, and the maturation of B lymphocytes. Note: Solid black arrows indicate activation or signaling pathways; dashed black arrows represent indirect or simplified signaling events; T-bars indicate inhibition.

NF-κB activation occurs via two pathways (as shown in Figure 2): the canonical (classical) pathway and the non-canonical (alternative) pathway. The canonical pathway is activated by various stimuli, including TNF, IL-1β, LPS, and ROS, which interact with their respective membrane receptors such as the TNF receptor (TNFR), the interleukin-1 receptor (IL-1R), and TLRs. Following receptor activation, adaptor proteins are recruited and activate the IKK complex, composed of the IKKα and IKKβ subunits and the essential NF-κB modulator (NEMO, also known as IKKγ).

This protein complex promotes the phosphorylation of the inhibitory protein IκB, particularly IκBα, directing it toward ubiquitination and degradation by the proteasomal system, which results in the release of NF-κB dimers. Finally, the dimers translocate to the nucleus, where gene expression is regulated through interaction with specific DNA sequences, known as κB sites, inducing the transcription of various genes related to the modulation of inflammation and the immune response [47,48].

The non-canonical pathway is activated by more specific stimuli, such as certain members of the TNF superfamily, including CD40 ligand (CD40L), B-cell activating factor (BAFF), and lymphotoxin beta (LTβ), which interact with their respective membrane receptors, such as the CD40 receptor (CD40R), the BAFF receptor (BAFF-R), and the lymphotoxin beta receptor (LTβR). The recruitment of adaptor proteins activates NF-κB-inducing kinase (NIK), which, by phosphorylating the IKKα subunit, promotes the phosphorylation of the p100 precursor. Subsequently, β-TrCP recognizes phosphorylated p100, leading to its ubiquitination and partial proteolytic processing, generating the p52 subunit. This subunit forms heterodimers with RelB that translocate to the nucleus, where they regulate genes related to adaptive immunity, lymphoid organ development, and B-cell maturation through interaction with specific DNA sequences (κB sites) [49,50].

NF-κB is one of the main regulators of the inflammatory response in the CNS due to its high constitutive expression, particularly in microglia and astrocytes [51,52]. Dysregulation of this pathway is associated with various neurodegenerative diseases such as Alzheimer’s disease (AD), related to the production of amyloid-β (Aβ) peptide (Sun2022); Parkinson’s disease (PD), where it participates in the inflammatory response and the death of dopaminergic neurons [53]; and epilepsy, by modulating neuronal excitability and the progression of epileptogenesis [54].

In the pathogenesis of epilepsy, NF-κB activation occurs primarily via the canonical pathway, with the response amplified due to crosstalk with other signaling pathways, such as MAPK, JAK/STAT, and PI3K/Akt, which promote sustained cytokine production, increased neuronal excitability, and alterations in synaptic plasticity. Studies using animal models demonstrate that, following epileptic seizures, there is hyperactivation of the pathway primarily in hippocampal neurons, glial cells, and endothelial cells in the CNS, which may persist for up to 8 days. NF-κB hyperactivation is commonly associated with blood–brain barrier dysfunction, glial activation, and neuronal death—factors that consolidate epileptogenesis and generate a positive feedback loop that sustains the neuroinflammation and neuronal excitability characteristic of epilepsy [55].

In this context, natural compounds are being investigated as potential new therapeutic options for epilepsy. Thus, (-)-epigallocatechin-3-gallate (EGCG) is a polyphenol extracted from Camellia sinensis (green tea) capable of inhibiting the TLR4/NF-κB signaling pathway and reducing IL-1β expression in rats treated with lithium-pilocarpine, with a reduction in synaptic dysfunction and neuronal loss in the hippocampus, as well as improved cognitive function (Table 2) [18,56].

Apigenin 6-C-glucoside-8-C-arabinoside (schaftoside) is a flavonoid found in plants used in traditional Chinese medicine, which has been shown to reduce apoptosis, the production of pro-inflammatory cytokines, and pentylenetetrazol (PTZ)-induced seizures in zebrafish (Table 2) by inhibiting the NF-κB pathway. Furthermore, it has been observed that this compound downregulates the expression of various interleukins, such as IL-6 and IL-1β [58].

Meanwhile, apigenin 7-O-neohesperidoside (rhoifolin) is a flavone found in lemons, bananas, and tomatoes, with evidence of preclinical efficacy as an antioxidant and anti-inflammatory agent in acquired epilepsy through inhibition of the NF-κB/iNOS/COX-2 axis in hippocampal neuronal culture assays. Studies have observed a reduction in apoptosis, oxidative stress, and levels of pro-inflammatory cytokines, indicating a possible neuroprotective effect in cases of acquired epilepsy (Table 2) [56].

Amentoflavone is a flavonoid found in over 120 plants that has demonstrated a reduction in the severity of epileptic seizures at a dose of 25 mg/kg in mice with pilocarpine-induced seizures (Table 2), through the reduction in pro-inflammatory cytokines and NF-κB [56,60]. Puerarin is a flavone extracted from Pueraria lobata (Ge Gen) that has been shown to reduce NF-κB mRNA expression in the hippocampus of rats with PTZ-induced epilepsy, in addition to acting as an antioxidant, suggesting a reduction in epileptogenic factors [18,63].

Furthermore, quercetin is a flavonoid found in fruits and vegetables that has been shown to inhibit NF-κB activation and help downregulate pro-inflammatory cytokines such as TNF, IL-1β, and IL-6. Some studies report that quercetin reduces the severity of seizures and repairs cellular damage in PTZ-induced epileptic rats (Table 2). Furthermore, quercetin decreases susceptibility to seizures in adulthood in rats with hypoxia-induced neonatal seizures, suggesting neuroprotective potential in the progression of epilepsy [56,61,64]. Thus, NF-κB represents an important target for the development of new compounds with antiepileptic effects.

### 2.3. MAPK

Mitogen-activated protein kinases (MAPKs) constitute a family of serine/threonine kinases involved in the regulation of proliferation, differentiation, and apoptosis in response to extracellular stimuli [65]. In basal conditions, MAPKs remain inactive and are activated by the interaction of extracellular stimuli with cell surface receptors and phosphorylation events, including growth factors, pro-inflammatory cytokines, and oxidative stress [66].

MAPK structures consist of amino acid chains organized into conserved domains responsible for catalytic activity and substrate recognition; they feature a characteristic kinase domain where specific threonine and tyrosine residues are phosphorylated, a process essential for their activation and function. In addition, specific structural regions allow interaction with other proteins in the signaling cascade, which ensures the accuracy and specificity of cellular responses [67].

MAPKs can be divided into three main subtypes: extracellular signal-regulated kinases (ERKs), c-Jun N-terminal kinases (JNKs), and p38 MAPKs. The ERK protein is involved in the regulation of cell proliferation, differentiation, and survival; it is typically activated by growth factors, and its dysregulation is strongly associated with cancer pathogenesis, since persistent activation of this pathway can lead to uncontrolled cell proliferation [68].

In contrast, JNK is predominantly activated in response to stress stimuli, such as pro-inflammatory cytokines, oxidative stress, and cellular damage, playing an important role in the regulation of apoptosis and the cellular stress response. Furthermore, by modulating intracellular signaling pathways, such as the NF-κB pathway, it leads to the production of pro-inflammatory cytokines, such as interleukin-1 (IL-1). Thus, it is involved in the pathophysiology of neurodegenerative diseases [66,69].

p38 MAPKs are also activated by stress and inflammatory stimuli and are directly involved in the production of pro-inflammatory cytokines such as IL-1 and TNF, in the infiltration of immune cells into tissues, and in apoptotic processes [70]. In general, activation of these pathways occurs through a sequential phosphorylation cascade involving three levels of kinases: MAP kinase kinase kinases (MAP3Ks), MAP kinase kinases (MAP2Ks), and, finally, MAPKs [68].

In the central nervous system, MAPKs play a fundamental role in the integration of chemical and electrical signals, regulating functions such as neuroplasticity, learning, and memory. Thus, dysregulation of these pathways is associated with pathological conditions, including neuroinflammatory disorders and neurological diseases, such as Alzheimer’s disease [71].

**Figure 3:**
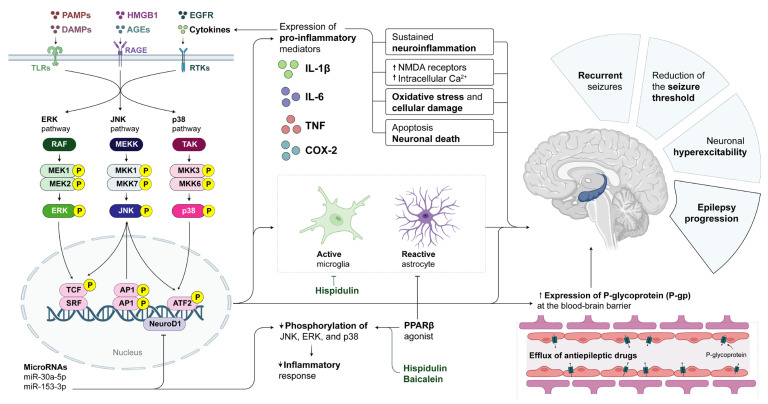
Schematic illustration of the role of MAPK signaling pathways (ERK, JNK, and p38) in the pathophysiology of epilepsy. Inflammatory stimuli, such as PAMPs, DAMPs, HMGB1, and AGEs, activate membrane receptors (TLRs, RAGE, and RTKs), triggering intracellular cascades involving RAF/MEK/ERK, MEKK/MKK/JNK, and TAK/MKK/p38. Activation of these pathways promotes the phosphorylation of transcription factors (TCF, SRF, AP-1, ATF2), modulating gene expression in the nucleus, including microRNAs. As a consequence, there is increased expression of pro-inflammatory mediators (IL-1β, IL-6, TNF, and COX-2), leading to the activation of reactive microglia and astrocytes, sustained neuroinflammation, oxidative stress, increased intracellular Ca^2+^, and excitotoxicity. These processes contribute to neuronal death, hyperexcitability, a lowered seizure threshold, and the progression of epilepsy. Additionally, increased expression of P-glycoprotein (P-gp) is observed in the blood–brain barrier, favoring the efflux of antiepileptic drugs. Compounds such as hispidulin and baicalein exert a modulatory effect by reducing MAPK phosphorylation and the inflammatory response, highlighting their therapeutic potential. Note: Solid black arrows indicate activation or signaling pathways; T-bars indicate inhibition.

Signals, such as indicators of cellular damage, interact with TLRs, which can be located both on the plasma membrane and in intracellular compartments, and this interaction triggers an intracellular signaling cascade that culminates in the activation of pathways such as MAPKs, promoting the expression of inflammatory mediators, as shown in Figure 3. Prolonged activation of these pathways contributes to the amplification of the inflammatory response and, in the long term, leads to the development of a neuroinflammatory state [72].

MAPKs are also involved in the neuroinflammatory mechanism through RAGE signaling. Generally, this pathway is activated by the binding of ligands such as advanced glycation end products (AGEs) and high mobility group box 1 (HMGB1). Upon activation, RAGE triggers an intracellular signaling cascade that leads to the activation of pathways such as MAPKs, as well as transcription factors such as NF-κB, resulting in increased expression of pro-inflammatory mediators, including pro-inflammatory cytokines and chemokines [72].

MAPKs are primarily regulated by dual-specificity phosphatases (DUSPs), particularly MKP-1. DUSPs play a key role as negative feedback regulators by dephosphorylating the MAPKs ERK, p38, and JNK. In models of chronic stress, hippocampal overexpression of MKP-1 results in reduced ERK/p38 phosphorylation, correlating with depressive-like behaviors, microglial activation, and elevated levels of the pro-inflammatory cytokines IL-6, IL-1β, and TNF [73]. Furthermore, the restoration of DUSP levels in damaged neurons and glia emerges as a neuroprotective strategy capable of counteracting the pro-apoptotic and pro-inflammatory hyperactivation of MAPKs [74].

Furthermore, the activity of these pathways is modulated by post-transcriptional regulators that modulate the immune response in the central nervous system. MicroRNAs such as miR-30a-5p and miR-153-3p act on the NeuroD1 target, inhibiting the phosphorylation of JNK, ERK, and p38 in microglia and astrocytes, resulting in the inhibition of NF-κB and NLRP3. Furthermore, signaling via peroxisome proliferator-activated receptors (PPARs) in astrocytes demonstrates potent regulatory control, as PPARβ agonists are capable of inhibiting LPS-induced phosphorylation of the MAPKs ERK, p38, and JNK [75].

In the context of epilepsy, intracellular signaling pathways play a key role in mediating processes associated with neuronal excitability, neuroinflammation, and cell death. Among these pathways, MAPKs have been extensively studied due to their involvement in regulating the molecular mechanisms underlying the pathophysiology of epileptic seizures [76]. JNK is widely distributed in the brain and is considered an important component in the regulation of processes such as gene expression; this kinase plays a significant role in the pathogenesis of epilepsy, such as in temporal lobe epilepsy and post-traumatic epilepsy [77]. JNK’s involvement occurs through the modulation of pathways related to excitotoxicity, inflammation, and cell death.

In vivo studies demonstrate that blocking specific isoforms or inhibiting JNK activity is associated with a reduction in the frequency and severity of seizures in temporal lobe epilepsy [78]. An in vivo study conducted in rats investigated the influence of the JNK pathway in a model of pilocarpine-induced seizures, focusing on the equilibrative nucleoside transporter 1 (ENT1), a transporter associated with the homeostasis of adenosine and glutamate levels—neurotransmitters critical to the development of epilepsy pathophysiology. The results demonstrated that, following epileptic seizures, there was a significant increase in JNK pathway activation, evidenced by its overexpression [79].

In physiological conditions, ERK stimulates the expression of NMDA receptors. A preclinical study identified the overexpression of glutamatergic receptors, as well as the activation of apoptotic mechanisms associated with death-associated protein kinase 1 (DAPK1), mediated by the ERK pathway. In the study, it was observed that inhibition of the ERK–DAPK1 axis resulted in antiapoptotic and anticonvulsant effects, compared to untreated groups, in which the opposite effect was observed. The results were consistent in both in vitro experiments and in vivo models [80].

p38 MAPK is associated with epilepsy through mechanisms related to the regulation of the expression of factors involved in purinergic signaling, including ENT1. In in vivo models, the influence of p38 on the modulation of adenosine receptor expression in the hippocampus and temporal lobe neocortex of rats was observed. Under epileptic conditions, overexpression of the adenosine A1 receptor (A1R) was observed in animals induced to have seizures. However, treatment with p38 inhibitors resulted in increased latency to seizure onset, as well as reduced neuronal damage, compared to untreated groups [81].

Concurrently, it has been observed that the integration of these pathways contributes to the establishment and maintenance of an epileptic state through the activation of various molecular mechanisms involving numerous transcription factors, proteins, and enzymes. In this context, MAPKs play a prominent role in both the modulation of neuroinflammation and the pathophysiology of epilepsy [76].

Given this, the search for therapeutic strategies capable of modulating these pathways has gained relevance, especially regarding the use of natural products. Several naturally derived compounds have demonstrated potential in regulating MAPK pathways and may exhibit anti-inflammatory, antioxidant, and neuroprotective properties. Among the classes of natural products that influence MAPKs in the context of epilepsy, flavonoids stand out as a significant class of bioactive compounds. Flavonoids are plant-derived phenolic compounds widely found in fruits, vegetables, and seeds, for example [82].

Among the flavonoids with the potential to modulate MAPK pathways in the context of epilepsy, several relevant bioactive compounds stand out. Among them, baicalein can regulate MAPK activity by modulating intracellular pathways associated with the inflammatory response (Table 3). As a result, a reduction in the expression of pro-inflammatory mediators is observed, contributing to the attenuation of neuroinflammation and the control of neuronal hyperexcitability, factors directly involved in the pathophysiology of epilepsy [83].

Another notable flavonoid is hispidulin, which has the ability to reduce microglial activation and the production of pro-inflammatory cytokines by modulating the MAPK pathway, particularly through the inhibition of enzymatic phosphorylation events. In experimental models of epilepsy, this property contributes to the control of the inflammatory process in the central nervous system and may be associated with a reduction in the frequency and intensity of epileptic seizures (Table 3) [83,84]. It has been observed that naturally occurring compounds represent a promising strategy for modulating MAPK signaling pathways, contributing to the attenuation of neuroinflammation and the control of processes associated with epileptogenesis.

In experimental models of refractory epilepsy, inhibition of p38 MAPK reduced the expression of the efflux transporters P-gp and multidrug resistance-associated protein 1, increased brain concentrations of antiepileptic drugs, and attenuated seizure severity [85,86]. These findings suggest that MAPK activation promotes drug resistance by limiting drug accumulation within the epileptic brain. Consistent with this hypothesis, patients with drug-resistant mesial temporal lobe epilepsy exhibit activation of the Ras/Raf/MAPK pathway, accompanied by increased ERK1/2 phosphorylation in epileptogenic tissue [87].

Given the central role of MAPK signaling in DRE, natural products capable of modulating this pathway have attracted growing interest. Numerous natural products, including alkaloids, flavonoids and terpenoids, present anticonvulsant activity through mechanisms involving glutamate and GABA systems, oxidative stress, and neuroinflammation [88,89]. Although direct evidence linking these compounds to reversal of pharmacoresistance is still lacking, computational studies have identified potential interactions with MAPK proteins. For example, bioactive constituents of Phyllanthus emblica, such as luteolin and kaempferol, showed strong predicted binding to MAPK1 and MAPK3 and were associated with pathways related to synaptic function, inflammation, and epileptogenesis [90]. Together, these findings suggest that MAPK-modulating natural products may represent promising candidates for overcoming DRE, although experimental validation is still required.

**Table 3:** Natural compounds evaluated in experimental models of epilepsy.

Natural Compound	Treatment Regimen	Experimental Model	References
Baicalein	10, 20, and 40 mg/kg/day, i.p.	In vivo (Genetic epilepsy-like tremor rats [TRM strain])	[91]
	In vivo: 20, 40, and 80 mg/kg/day, oral administration. In vitro: 5, 10, and 20 μM	In vivo: Sprague-Dawley rats, Lithium-Pilocarpine-induced SE model In vitro: BV2 microglia, LPS-stimulated (100 ng/mL)	[92]
Hispidulin	3, 10, 30, and 100 μM	In vitro (BV2 microglia, LPS-stimulated: 100 ng/mL)	[84]

### 2.4. mTOR

The mTOR signaling pathway (mammalian Target of Rapamycin) has been widely recognized as a fundamental integrative axis linking metabolic signals, cellular growth, synaptic plasticity, and immune responses within the central nervous system. Its relevance in the context of epilepsy arises from the convergence of structural alterations, synaptic dysfunctions, and inflammatory processes that characterize epileptogenic brain tissue [93,94].

From a molecular perspective, mTOR is a serine/threonine kinase belonging to the phosphatidylinositol-3-kinase-related kinase (PIKK) family, whose activity is organized into two functionally distinct complexes: mTORC1 and mTORC2. mTORC1, which is rapamycin-sensitive, regulates processes such as protein synthesis, ribosomal biogenesis, lipid metabolism, and inhibition of autophagy, whereas mTORC2 is involved in cytoskeletal organization, cell survival, and activation of pathways such as Akt [95].

**Figure 4:**
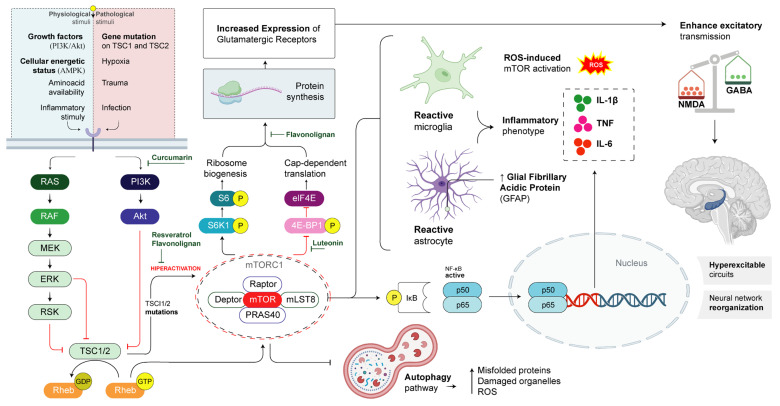
mTOR signaling pathway. Genetic triggers (such as mutations in *TSC1*/*TSC2*) or acquired triggers (trauma, hypoxia, status epilepticus) lead to a loss of inhibitory control over the mTOR pathway, resulting in hyperactivation of the mTORC1 complex. This activation promotes increased protein synthesis and dysregulated cell growth, culminating in structural brain alterations, such as cortical dysplasia, dysmorphic neurons, and defects in neuronal migration. Concurrently, synaptic remodeling occurs, characterized by increased glutamatergic excitability and impaired GABAergic inhibition, establishing an excitation/inhibition imbalance. This scenario favors neuronal hyperexcitability, the formation of epileptogenic networks, and the emergence of recurrent spontaneous seizures. Seizures induce neuroinflammation, oxidative stress, mitochondrial dysfunction, and reduced autophagy—mechanisms that feed back into the activation of the mTOR pathway, perpetuating a self-sustaining pathological cycle. Note: Solid black arrows indicate activation or signaling pathways; red T-bars indicate inhibition.

Activation of the mTOR pathway, as illustrated in Figure 4, is modulated by multiple extracellular and intracellular signals, including growth factors (PI3K/Akt), cellular energetic status (AMPK), amino acid availability, and inflammatory stimuli. Under physiological conditions, this regulation is finely tuned; however, in several pathological contexts, including structural and genetic epilepsies, persistent and dysregulated activation of the pathway is observed. This hyperactivation may result from mutations in regulatory genes such as *TSC1* and *TSC2*, or from secondary stimuli following brain insults such as trauma, hypoxia, or infection [96].

Within the epileptic brain microenvironment, the mTOR pathway plays a central role in modulating neuroinflammation. Activation of mTOR in glial cells, particularly astrocytes and microglia, promotes their transition into a reactive state characterized by morphological and functional changes. Reactive astrocytes exhibit increased expression of GFAP, altered regulation of glutamate and extracellular potassium homeostasis, and secretion of inflammatory mediators. Activated microglia, in turn, adopt a predominantly pro-inflammatory phenotype, releasing cytokines such as IL-1β, TNF, and interleukin-6 (IL-6), as well as chemokines and reactive oxygen species [97].

The regulation of these inflammatory responses by mTOR involves, in part, interaction with classical signaling pathways such as the NF-κB pathway, one of the main transcriptional regulators of pro-inflammatory gene expression. mTORC1 activation can modulate NF-κB activity both directly and indirectly, influencing the translation of proteins involved in the inflammatory cascade and promoting amplification of the innate immune response in the central nervous system. This process contributes to the maintenance of a chronic low-grade inflammatory state, which has been associated with reduced seizure threshold and facilitated propagation of epileptiform discharges [95,98].

In parallel, mTOR hyperactivation impacts the integrity of the blood–brain barrier (BBB). Alterations in the expression of tight junction proteins such as claudins and occludins lead to increased vascular permeability, allowing extravasation of plasma proteins such as albumin and infiltration of peripheral immune cells. The presence of these elements within the brain parenchyma triggers additional inflammatory responses, including astrocyte activation via receptors such as TGF-β, thereby reinforcing the inflammatory cycle and contributing to epileptogenesis [94].

Another critical component mediated by the mTOR pathway is autophagy regulation. Under conditions of mTORC1 hyperactivation, this catabolic process is inhibited, resulting in the accumulation of misfolded proteins, damaged organelles, and increased oxidative stress. Autophagy impairment has been associated with synaptic dysfunction and inflammatory activation, as the inadequate removal of dysfunctional cellular components may act as a danger signal, triggering intracellular immune responses [98].

At the neuronal level, the mTOR pathway contributes to alterations in excitability and neural network organization. mTORC1 activation promotes increased synthesis of synaptic proteins, including glutamatergic receptors and components of neurotransmitter release machinery, thereby enhancing excitatory transmission. In addition, there is evidence that mTOR participates in aberrant axonal sprouting, such as mossy fiber sprouting in the hippocampus, frequently observed in temporal lobe epilepsy. This structural reorganization contributes to the formation of reverberating and hyperexcitable circuits, facilitating seizure generation and propagation [94,96,97].

The dynamic interaction between neuronal activity, inflammation, and mTOR-mediated metabolic signaling forms a feedback loop in which epileptic seizures induce inflammatory and mTOR activation, while this activation in turn perpetuates structural and functional alterations that favor further seizures. This model reinforces the understanding of epilepsy as a multifactorial disorder in which immunological and metabolic processes are as relevant as classical electrophysiological abnormalities [98]. Recently, there has been a growing interest in natural products targeting the mTOR pathway in epilepsy, driven by its central role in epileptogenesis. Plant-derived compounds such as polyphenols and flavonoids are being explored for their ability to modulate neuroinflammation, neuronal excitability, and synaptic plasticity through multiple molecular targets.

Among the natural products, resveratrol is the compound with some of the most consistent evidence of direct modulation of the mTOR pathway in epilepsy models. In an experimental study, it was demonstrated that resveratrol pre-treatment significantly reduces mTOR activation following status epilepticus, as well as attenuates the expression of inflammatory mediators such as IL-1β, COX-2, and iNOS. These effects were associated with AMPK activation, indicating an upstream mechanism of mTOR inhibition [38].

Silibinin, a flavonolignan derived from Silybum marianum, has been investigated as a modulator of intracellular pathways involved in neuroinflammation and neuronal excitability, including the mTOR pathway. Experimental data indicate that this compound may reduce mTORC1 activation, with decreased phosphorylation of downstream targets such as p70S6K and 4E-BP1, suggesting functional inhibition of this pathway (Table 4). These findings are relevant in the context of epileptogenesis, since mTOR hyperactivation is associated with processes such as aberrant synaptic reorganization, neuroinflammation, and increased neuronal excitability, although direct evidence in experimental epilepsy models remains limited [18,99].

Moreover, curcumin, the main polyphenol extracted from Curcuma longa, has been extensively investigated for its anti-inflammatory and neuroprotective effects, with potential relevance in neurological conditions associated with hyperactivation of the mTOR pathway. Experimental evidence indicates that curcumin may indirectly modulate this pathway, primarily through AMPK activation and inhibition of PI3K/Akt signaling, leading to functional suppression of mTORC1 activity. These effects contribute to the reduction in processes involved in epileptogenesis, such as neuroinflammation, oxidative stress, and glutamatergic excitotoxicity [103].

Finally, the flavonoid Luteolin exhibits neuroprotective and antiepileptic effects in experimental models, mainly by reducing neuroinflammation, oxidative stress, and excitotoxicity (Table 4). Among its multiple mechanisms, modulation of intracellular pathways related to epileptogenesis is notable, including the mTOR pathway. Studies indicate that luteolin may inhibit activation of the mTOR/4E-BP1 pathway in neuronal cells, contributing to reduced excitability and seizure susceptibility [64].

### 2.5. COX-2/PGE_2_

**Figure 5:**
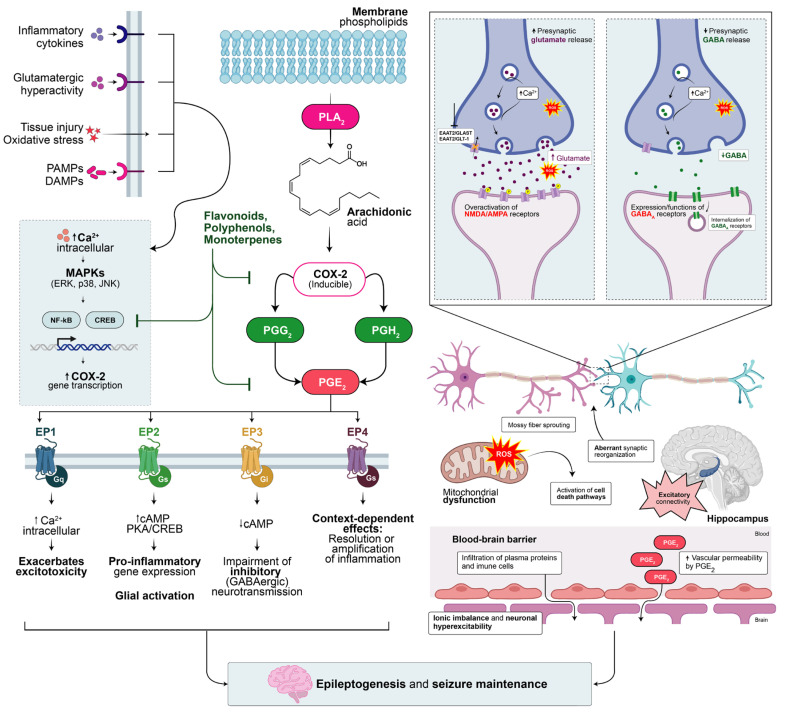
Molecular mechanisms of the COX-2/PGE_2_ in epilepsy. In epileptic conditions, inflammatory stimuli (e.g., cytokines, glutamate, LPS/PAMPs/DAMPs) trigger microglial activation and neuronal hyperexcitability, leading to phospholipase A_2_ (PLA_2_)-mediated release of arachidonic acid from membrane phospholipids. Arachidonic acid is subsequently metabolized by inducible cyclooxygenase-2 (COX-2) to generate prostaglandin E_2_ (PGE_2_), a key mediator that signals through EP1–EP4 receptors to regulate intracellular Ca^2+^ dynamics, cAMP/PKA/CREB pathways, and pro-inflammatory gene expression. This cascade contributes to hallmark features of epilepsy, including excitotoxicity, sustained neuroinflammation, synaptic dysfunction, blood–brain barrier disruption, and structural remodeling, ultimately driving epileptogenesis and seizure maintenance. Note: Solid black arrows indicate activation or signaling pathways; T-bars indicate inhibition.

The cyclooxygenase-2 (COX-2) and arachidonic acid pathway constitutes one of the principal enzymatic systems responsible for the generation of bioactive lipid mediators both in peripheral tissues and in the central nervous system. Activation of this pathway (Figure 5) begins with the release of arachidonic acid from membrane phospholipids, catalyzed by phospholipase A_2_ (PLA_2_) in response to physiological and inflammatory stimuli, leading to increased intracellular Ca^2+^ levels and activation of pharmacological receptors. Once released, arachidonic acid is converted by COX-2 into prostaglandin G2 (PGG_2_) and subsequently into prostaglandin H_2_ (PGH_2_), which serves as a precursor for various prostanoids, including prostaglandin E_2_ (PGE_2_), a key regulator of inflammation, pain, and fever. Unlike COX-1, which is constitutively expressed and associated with homeostatic functions, COX-2 is highly inducible, with its expression regulated by inflammatory, excitatory, and injurious stimuli, being particularly relevant in pathological contexts such as epilepsy [104,105].

In the central nervous system, COX-2 induction occurs predominantly in excitatory neurons following events of synaptic hyperactivity, such as epileptic seizures. Excessive activation of NMDA and AMPA receptors promotes Ca^2+^ influx, triggering intracellular signaling cascades that include protein kinases (MAPKs) and transcription factors such as NF-κB and CREB, ultimately culminating in COX-2 overexpression. This enzymatic upregulation leads to excessive PGE_2_ production, which acts as a potent modulator of neuronal excitability and inflammatory responses, establishing a direct link between abnormal electrical activity and neuroinflammation [106,107].

PGE_2_ exerts its effects through four receptor subtypes (EP1–EP4), each activating distinct intracellular pathways and eliciting specific cellular responses. Activation of the EP1 receptor is associated with increased intracellular Ca^2+^, exacerbating neuronal excitotoxicity. The EP2 receptor activates the cAMP/PKA/CREB pathway, promoting the expression of pro-inflammatory genes and sustaining glial activation. EP3, by reducing cAMP levels, may impair inhibitory neurotransmission, whereas EP4 exhibits more complex effects, contributing either to the resolution or amplification of inflammation depending on the cellular context [106].

In the pathophysiology of epilepsy, activation of the COX-2/PGE_2_ pathway leads to dysregulation of glutamatergic neurotransmission. PGE_2_ enhances presynaptic glutamate release by facilitating Ca^2+^ influx at nerve terminals and modulating proteins involved in vesicular exocytosis. Concurrently, there is a reduction in the expression and function of astrocytic glutamate transporters (EAAT1/GLAST and EAAT2/GLT-1), impairing neurotransmitter clearance from the synaptic cleft. Consequently, extracellular glutamate accumulates, leading to overactivation of NMDA and AMPA receptors, intracellular Ca^2+^ overload, activation of degradative enzymes, and increased production of reactive oxygen species, thereby characterizing a state of sustained excitotoxicity [104,108,109].

In parallel, the COX-2/PGE_2_ pathway compromises GABAergic neurotransmission, contributing to the imbalance between excitation and inhibition. PGE_2_ reduces GABA release from inhibitory interneurons and may negatively modulate the expression of postsynaptic GABA_A_ receptors. Additionally, pro-inflammatory cytokines such as IL-1β and TNF, whose expression is induced by COX-2 activation, promote internalization of GABA_A_ receptors and increase phosphorylation of glutamatergic receptors, further amplifying neuronal excitability. Collectively, these alterations favor the establishment and maintenance of hyperexcitable neuronal circuits characteristic of epilepsy [104,108].

Another critical aspect of the COX-2/PGE_2_ pathway is its impact on the integrity of the BBB. PGE_2_ increases vascular permeability, allowing the infiltration of plasma proteins and inflammatory cells into the brain parenchyma. The presence of albumin within brain tissue activates TGF-β-dependent signaling pathways in astrocytes, disrupting ionic homeostasis and promoting neuronal hyperexcitability. Moreover, sustained microglial activation intensifies the release of inflammatory mediators and reactive species, perpetuating the cycle of neuroinflammation [110].

Chronic activation of this pathway is also associated with structural alterations that sustain epileptogenesis, including mossy fiber sprouting, aberrant synaptic reorganization, and increased excitatory connectivity in the hippocampus. These processes are accompanied by mitochondrial dysfunction, oxidative stress, and activation of cell death pathways, contributing to disease progression [106,111].

Within this pathophysiological framework, it is important to highlight that natural compounds have emerged as promising modulators of the COX-2 pathway. Studies with rhoifolin, a natural flavonoid, have shown in in vitro tests of acquired epilepsy using the HT-22 cell line that different doses of the compound (5, 10, and 20 µM) increased the recovery of cell viability and reduced markers of neuronal damage, with peak efficacy observed at a dose of 20 µM. In addition, Western blot analysis demonstrated that treatment with rhoifolin decreased levels of p-p65, p-IκBα, iNOS, and COX-2, which were elevated in the epileptic groups [59].

From a therapeutic perspective, these findings reinforce that selective modulation of the COX-2 pathway, particularly by natural compounds, represents a promising strategy for controlling neuroinflammation and neuronal excitability. However, it is important to emphasize the need for studies on new compounds that act through the COX pathway for the treatment of epilepsy.

### 2.6. TLR4/HMGB1

High Mobility Group Box 1 (HMGB1) is a 215-amino acid protein belonging to the HMG (High Mobility Group) family. Structurally, it is characterized by two DNA-binding domains, the Box A and Box B domains, followed by an acidic tail at the C-terminal end [112]. Functionally, HMGB1 acts in two ways: at the intracellular level, it modulates transcription and gene regulation; in its extracellular form, it behaves as a pro-inflammatory cytokine, playing a crucial role in the inflammatory response [113].

Extracellular HMGB1, particularly in its disulfide isoform, acts as a central mediator in the CNS’s innate immunity by interacting with the TLR4. This receptor is widely distributed on the surface of neurons, astrocytes, and microglia, playing a key role in the detection of DAMPs [114]. The binding of HMGB1 to TLR4 triggers intracellular signaling cascades dependent on the adaptor protein MyD88, culminating in the translocation of transcription factors such as NF-κB and the activation of MAPK pathways [115].

**Figure 6:**
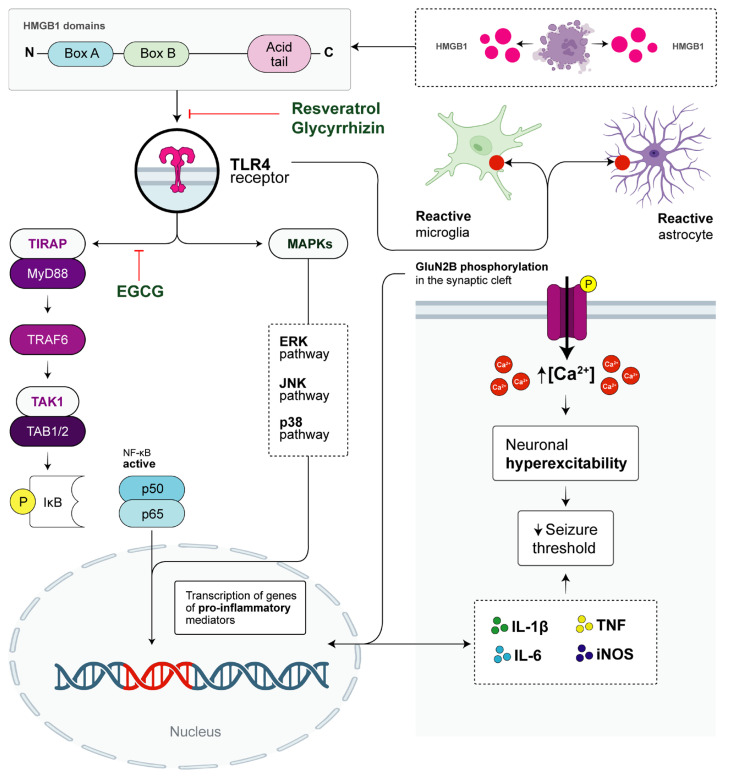
Schematic representation of the HMGB1/TLR4 pathway associated with neuroinflammation and epileptogenesis. Extracellular release of the HMGB1 protein promotes activation of the TLR4 receptor, triggering MyD88/TRAF6/TAK1- and MAPK-dependent signaling pathways, culminating in NF-κB activation and the transcription of pro-inflammatory mediators. The activation of reactive microglia and astrocytes promotes the production of inflammatory cytokines, such as IL-1β and TNF. Concurrently, phosphorylation of the GluN2B subunit of the NMDA receptor increases Ca^2+^ influx and neuronal hyperexcitability, lowering the seizure threshold. Compounds such as resveratrol, glycyrrhizin, and EGCG act as inhibitory modulators of this inflammatory pathway. Note: Solid black arrows indicate activation or signaling pathways; red T-bars indicate inhibition.

This interaction, which occurs in neurons and glial cells (microglia and astrocytes), triggers MyD88-dependent signaling cascades, culminating in the activation of transcription factors such as NF-κB and the release of pro-inflammatory cytokines, such as IL-1β [113]. In the synaptic environment, this axis promotes phosphorylation of the GluN2B subunit of NMDA receptors, exacerbating the intracellular influx of calcium (Ca^2+^) and lowering the seizure threshold, as illustrated in Figure 6 [113,115].

Thus, a positive feedback loop is established, where the hyper-excitability itself stimulates the expression and translocation of HMGB1, perpetuating damage and neuroinflammation [113]. Furthermore, TLR4 has been associated with the regulation of autophagy processes in inhibitory (GABAergic) neurons, where its dysfunction can exacerbate the imbalance between excitation and inhibition in the brain [115,116].

Elevated levels of HMGB1 and TLR4 have been consistently associated with more severe forms of epilepsy, reinforcing their potential as markers of disease progression. In patients with drug-resistant epilepsy, a significant increase in the concentrations of these proteins is observed in both serum and cerebrospinal fluid, compared to individuals responsive to conventional antiepileptic therapy [117]. Furthermore, these findings are supported by the existence of a positive linear correlation between serum levels of HMGB1 and TLR4 and clinical parameters of the disease, including the frequency and duration of epileptic seizures [118], suggesting the active involvement of these molecules in modulating clinical severity.

Furthermore, the activation of this molecular pathway is directly related to the overexpression of P-gp, an important efflux pump located in the blood–brain barrier, which compromises the cerebral bioavailability of antiepileptic drugs by promoting their active efflux out of the CNS, preventing them from reaching adequate therapeutic concentrations and thus contributing to the development of drug resistance [113,118].

Several studies have investigated the potential of natural products as modulators of the HMGB1/TLR4 axis, with the aim of attenuating neuroinflammation and, consequently, reducing epileptic seizures. Glycyrrhizin (GL), also known as glycyrrhizic acid, is a low-molecular-weight compound classified as a glycopyranosiduronic acid and structurally defined as an oleanane-type triterpenoid saponin, predominantly isolated from the roots of Glycyrrhiza glabra [113,119].

This metabolite is among the best-characterized natural inhibitors of HMGB1. At the molecular level, GL interacts directly with HMGB1 and HMGB2, inhibiting their phosphorylation by kinases such as CK-I and PKC, which results in a reduction in their ability to bind to DNA and, consequently, in the attenuation of the inflammatory response. Additionally, its mechanism of action involves binding to the functional domains (Box A and B) of HMGB1, blocking its extracellular activity and modulating its neuronal release [119].

Glycyrrhizin has demonstrated significant anticonvulsant and neuroprotective effects in experimental models of epilepsy. Treatment significantly increased seizure latency and reduced seizure duration in a dose-dependent manner, with 50 mg/kg/day identified as the most effective dose (Table 4). In addition to improving seizure-related parameters, glycyrrhizin attenuated neuronal damage, reducing neuronal loss in the CA1 and CA3 regions of the hippocampus and preserving histological integrity, resulting in a higher number of surviving neurons compared to untreated animals. From a mechanistic perspective, these effects were associated with inhibition of the HMGB1/TLR4/NF-κB signaling pathway, leading to reduced activation of pro-inflammatory cascades. Consequently, glycyrrhizin significantly decreased the production of the pro-inflammatory cytokines IL-1β and IL-18 in both the hippocampus and serum, thereby mitigating the chronic neuroinflammatory response associated with epileptogenesis [102].

Resveratrol has been extensively studied for its neuroprotective properties, with potential effects observed across various pathways. Studies have observed a reduction in the release of HMGB1 induced by oxidative stress [112]. Notably, nanotechnological formulations, such as nanoresveratrol, exhibit greater bioavailability and efficacy, promoting the inhibition of HMGB1 and TLR4 expression in critical regions such as the hippocampus [39]. These effects translate into the attenuation of acute epileptic seizures, as well as the mitigation of neuronal damage and subsequent cognitive deficits.

Another notable compound is epigallocatechin-3-gallate (EGCG), the main catechin in green tea derived from Camellia sinensis. This polyphenol exerts potent anti-inflammatory and anti-apoptotic effects by inhibiting the TLR4/NF-κB signaling pathway, one of the main regulatory axes of the inflammatory response in the central nervous system. In experimental models based on kindling, EGCG has demonstrated the ability to slow the progression of epilepsy, while simultaneously reducing chronic neuroinflammation [114]. These compounds collectively demonstrate the potential of natural products as modulators of the HMGB1–TLR4 axis, offering promising prospects for the development of innovative therapeutic approaches aimed at controlling epilepsy and its neuroinflammatory complications.

## 3. Conclusions

In conclusion, accumulating evidence indicates that neuroinflammatory and cell-signaling pathways play central roles in the pathophysiology of epilepsy, contributing to neuronal hyperexcitability, neurodegeneration, and disease progression. Key molecular targets, including the NLRP3 inflammasome, NF-κB, MAPK, mTOR, COX-2/PGE2, and TLR4/HMGB1 pathways, are closely associated with seizure generation, epileptogenesis, and pharmacoresistance. In this context, natural compounds have emerged as promising therapeutic candidates due to their ability to simultaneously modulate multiple pathogenic mechanisms. Compounds such as fisetin, curcumin, resveratrol, EGCG, quercetin, glycyrrhizin, baicalein, hispidulin, luteolin, and silibinin have demonstrated anticonvulsant, anti-inflammatory, antioxidant, and neuroprotective properties in experimental models of epilepsy through the regulation of these signaling pathways. Notably, their multitarget actions may offer advantages over conventional antiseizure medications, which primarily provide symptomatic control and are often associated with adverse effects and treatment resistance.

Despite the encouraging preclinical evidence, several translational challenges remain before these compounds can be incorporated into clinical practice. Limitations related to bioavailability, blood–brain barrier permeability, pharmacokinetic variability, standardization of natural product formulations, and the scarcity of well-designed clinical trials continue to hinder their therapeutic development. Future research should focus on elucidating the precise molecular mechanisms underlying their neuroprotective effects, optimizing delivery systems and dosing strategies, identifying reliable biomarkers of neuroinflammation, and evaluating long-term efficacy and safety in patients with epilepsy. Furthermore, greater efforts are needed to bridge the gap between experimental findings and clinical applications through translational studies and randomized controlled trials. Collectively, these advances may pave the way for the development of innovative anti-inflammatory therapies and natural product-based interventions capable not only of suppressing seizures but also of modifying the underlying processes of epileptogenesis and disease progression.

## Figures and Tables

**Table 1:** Natural compounds evaluated in experimental models of epilepsy.

Natural Compound	Treatment Regimen	Experimental Model	References
Fisetin	5 mg/kg/day (free form and nanoformulation, oral administration) for 28 days	In vivo (male BALB/c mice, Pilocarpine-induced temporal lobe epilepsy)	[36]
Curcumin	200 mg/kg/day, (oral administration) for 14 days	In vivo (Rats, PTZ-induced kindling model: 50 mg/kg, i.p.)	[37]
Resveratrol	40 mg/kg, i.p.	In vivo (Male Wistar rats, pilocarpine-induced SE model: 300 mg/kg, i.p.)	[38]
	Resveratrol: 40 mg/kg; Nanoresveratrol: 0.04, 0.4, and 4 mg/kg, i.p.	In vivo (Male Swiss albino mice, PTZ-induced [60 mg/kg, i.p.] and ICES models)	[39]
Neferine	10 and 50 mg/kg, i.p.	In vivo (Male Sprague-Dawley rats, KA-induced seizure model: 15 mg/kg, i.p.)	[40]

**Table 2:** Natural compounds evaluated in experimental models of epilepsy.

Natural Compound	Treatment Regimen	Experimental Model	References
Epigallocatechin-3-gallate (EGCG)	20 mg/kg/day, (oral administration) for 3 weeks	In vivo (Sprague-Dawley rats, chronic PTZ-induced kindling model)	[57]
Apigenin 6-C-glucoside-8-C-arabinoside	100, 200, and 400 μM	In vivo (Zebrafish larvae, PTZ-induced seizure model: 5 mM)	[58]
Rhoifolin	5, 10, and 20 μM	In vitro (HT-22 cells, MgCl2-free AE model)	[59]
Amentoflavone	25 mg/kg, p.o. (oral administration, pre or post-SE)	In vivo (Mice, pilocarpine-induced SE model: 300 mg/kg, i.p.)	[60]
Quercetin	In vivo: 100 mg/kg/day, i.p. In vitro: 10 nM	In vivo: BALB/c mice, KA-induced seizure (10 mg/kg, i.p.); In vitro: Primary mouse microglia, KA-stimulated (100 µM)	[61]
	5–200 mg/kg (dose–response) and 200–800 mg/kg (in combination with AEDs), i.e., i.p.	In vivo (Mice, 6 Hz psychomotor seizure model)	[62]

**Table 4:** Natural compounds evaluated in experimental models of epilepsy.

Natural Compound	Treatment Regimen	Experimental Model	References
Silibinin	50, 100, and 200 mg/kg	In vivo (C57BL/6 mice, intrahippocampal KA-induced seizure model)	[99]
Luteolin	10 and 50 mg/kg, i.p.	In vivo (Male Sprague-Dawley rats, KA-induced seizure model: 15 mg/kg, i.p.)	[100]
	120 mg/kg/day, (oral administration)	In vivo (C57BL/6 mice, chronic LPS-induced neuroinflammation model)	[101]
Glycyrrhizin	25, 50, and 100 mg/kg/day, i.p.	In vivo (C57BL/6J mice, intrahippocampal [CA3] KA-induced SE model)	[102]

## Data Availability

The original contributions presented in this study are included in the article. Further inquiries can be directed to the corresponding author.

## Abbreviations

The following abbreviations are used in this manuscript:

AA	Arachidonic acid
AD	Alzheimer’s Disease
A1R	Adenosine A1 Receptor
AMPK	Adenosine Monophosphate-Activated Protein Kinase
Aβ	Amyloid-Beta
BAFF	B-cell Activating Factor
BBB	Blood–Brain Barrier
cAMP	Cyclic Adenosine Monophosphate
CAT	Catalase
CD40	CD40 Receptor
CD40L	CD40 Ligand
CK-1	Casein Kinase 1
CNS	Central Nervous System
COX-1	Cyclooxygenase-1
COX-2	Cyclooxygenase-2
CREB	cAMP response element-binding protein
DAMPs	Damage-associated molecular patterns
DAPK1	Death-Associated Protein Kinase 1
DNA	Deoxyribonucleic Acid
DUSPs	Dual-Specificity Phosphatases
EGCG	Epigallocatechin-3-gallate
ENT1	Equilibrium nucleoside transporter 1
ERK	Extracellular Signal-Regulated Kinase
GABA	Gamma-Aminobutyric Acid
GFAP	Glial Fibrillary Acidic Protein
GL	Glycyrrhizin
GPx	Glutathione Peroxidase
GSH	Reduced Glutathione
HMGB1	High Mobility Group Box 1
IKK	IκB kinase
IL-1β	interleukin-1 beta
IL-6	interleukin-6
iNOS	Inducible Nitric Oxide Synthase
IκB	Inhibitor of κB
JAK/STAT	Janus Kinase/Signal Transducer and Activator of Transcription
JNKs	c-Jun N-terminal kinases
LPS	Lipopolysaccharide
LTβ	Lymphotoxin beta
MAP2Ks	MAP Kinase Kinases
MAP3Ks	MAP Kinase Kinase Kinases
MAPK	Mitogen-Activated Protein Kinase
MKP-1	Mitogen-Activated Protein Kinase Phosphatase 1
MS	Multiple Sclerosis
mTOR	mammalian Target of Rapamycin
MyD88	Myeloid Differentiation Primary Response 88
NEMO/IKKγ	NF-κB Essential Modulator/Inhibitor of κB Kinase gamma
NF-κB	Nuclear Factor-Kappa B
NIK	NF-κB-Inducing Kinase
NLR	Nod-Like Receptor
NLRP3	Nod-Like Receptor Family Pyrin Domain Containing 3
NMDA	N-Methyl-D-Aspartate
Nrf2	Nuclear Factor Erythroid 2–Related Factor 2
PAMPs	Pathogen-Associated Molecular Patterns
PGE_2_	Prostaglandin E_2_
PGG_2_	Prostaglandin G_2_
PGH_2_	Prostaglandin H_2_
P-gp	P-glycoprotein
PI3K	Phosphoinositide 3-Kinase
PIKK	Phosphatidylinositol 3-Kinase-Related Kinases
PKA	Protein Kinase A
PKB	Protein Kinase B
PKC	Protein Kinase C
PLA_2_	Phospholipase A_2_
PPARs	Peroxisome Proliferator-Activated Receptors
PRR	Pattern Recognition Receptor
RAGE	Receptor for Advanced Glycation End Products
RelB	v-rel Avian Reticuloendotheliosis Viral Oncogene Homolog B
RHD	Rel Homology Domain
ROS	Reactive Oxygen Species
SIRT1	Sirtuin 1
TAD	Transactivation Domain
TGF-β	Transforming Growth Factor Beta
TLR	Toll-Like Receptor
TNF	Tumor Necrosis Factor
TPA	12-O-tetradecanoylphorbol-13-acetate
TSC1	Tuberous Sclerosis Complex 1
TSC2	Tuberous Sclerosis Complex 2
β-TrCP	Beta-Transducin Repeat-Containing Protein
